# Molecular Biological Determinations of Meningioma Progression and Recurrence

**DOI:** 10.1371/journal.pone.0094987

**Published:** 2014-04-10

**Authors:** Stefan Linsler, Dennis Kraemer, Christina Driess, Joachim Oertel, Kai Kammers, Jörg Rahnenführer, Ralf Ketter, Steffi Urbschat

**Affiliations:** 1 Department of Neurosurgery, Saarland University, Homburg/Saar, Germany; 2 Department of Statistics, TU Dortmund University, Dortmund, Germany; 3 Department of Biostatistics, Johns Hopkins Bloomberg School of Public Health, Baltimore, Maryland, United States of America; University of Southern California, United States of America

## Abstract

Meningiomas are tumors that arise from the coverings of the brain or spinal cord. 5% of the cases turn into malignant forms with aggressive clinical behavior and increased risk of tumor recurrence. One hundred and five patients with meningiomas were operated by open surgery. To investigate predictors of meningioma recurrence in total 124 samples of 105 patients were investigated by iFISH. Dual-probe hybridization was performed to access chromosomal alterations of chromosomes 1p-, 9p- and 22q. Additionally, methylation of *TIMP3* and *p16* was analyzed with MS-PCR. Of the 105 investigated tumors 59.1% (62/105) were WHO grade I, 33.3% (35/105) were WHO grade II and 7.7% (8/105) were anaplastic meningiomas (grade III), respectively. The histopathological data correlates with the recurrence rate of the investigated meningiomas. Hypermethylation of *TIMP3* was detected in 13.3% of all meningiomas: 10.9% in WHO grade I meningiomas, 25.0% in grade II and 14.3% in grade III meningiomas, respectively. No correlation of *TIMP3* hypermethylation with tumor recurrence or WHO grade (p = 0.2) was observed. Interestingly, deletion of 1p36 emerged as a significant predictor of shorter overall survival (log rank test, *p*<0.001), whereas *TIMP3* promoter methylation had no significant effect on overall survival (log rank test, *p* = 0.799). The results of the current study support the finding that the deletion of chromosome 1p is an independent marker of meningioma recurrence and progression (p = 0.0097). Therefore the measurement of genetic aberrations in meningiomas allows in a combined histological approach a more precise assessment of the prognosis of meningiomas than histopathology alone.

## Introduction

Meningioma is a tumor composed of neoplastic meningothelial cells and accounts for 13 to 26 percent of intracranial tumors. In its sporadic form, it is typically benign and slow growing, appearing mostly in the later decades of life. Histological features allows for the division of meningiomas into three grades: benign meningiomas (WHO grade I), atypical meningiomas (WHO grade II) which represent 4.7 to 7.2 percent of all meningiomaş and anaplastic meningiomas (WHO grade III) representing 1.0 to 2.8 percent of all meningiomas. However, more than eight percent of all meningiomas are characterized by aggressive clinical behavior with increased risk of tumor recurrence [1]–[5].

Conventional cytogenetic analysis of meningiomas often reveals the frequent entire or partial losses of chromosome 22. Extensive studies by loss of heterozygosity (LOH) also have shown frequent allelic losses on the long arm of this chromosome, pinpointing the tumor suppressor locus in the vicinity of the *NF-2* gene [6]. This is supported by the fact that patients with type II neurofibromatosis, who have constitutional mutations of the *NF2* gene on the long arm of chromosome 22 (22q12.2), often present with multiple meningiomas. However this hereditary alteration has little or no influence on progression [7], [8]. A second tumor suppressor gene on chromosome 22 on 22q12.3 is the gene for the tissue inhibitor of metalloproteinase 3 (*TIMP3*), which appears to be involved in meningioma progression and a high-grade meningioma phenotype [9], [10]. *TIMP3* hypermethylation seems to be associated with the allelic loss of 22q12. Moreover, hypermethylation of the *TIMP3* promoter has been identified as a common reason of decreased *TIMP3* expression levels in numerous tumors like secondary glioblastomas, kidney cancer, or pancreatic adenocarcinomas [11], [12], [13], [14], [15]. As to intracranial tumors, methylation of MGMT in gliomas [16] and of *TIMP3* [9] and *p16* [17], [18] in meningiomas seems to be associated with aggressive tumor behavior.

The p16 pathway, which has been found to be altered in more than 80% of human cancers [17], [34], [35], is located on 9p12, one of the investigated chromosomal regions investigated in this present study by FISH [36]. It has recently been proven that hypermethylation of a normally unmethylated CpG island in the promoter region of p16 correlates with its loss of transcription in various primary cancers [15], [36]–[39]. Other studies of the p16 gene have found homozygous deletions, mutations or transcriptional inhibition by methylation in a large number of different human tumor types [40]–[42].

In contrast to other solid tumors, progression of meningiomas is correlated with increasing hypodiploidy, showing characteristic clonal evolutions that mostly include chromosomes 14,18, and 19 and, more rarely, 6 and 10. Structural aberrations are rare, except for the loss of the short arm of one chromosome 1, which appears to be the decisive step for anaplastic growth [3], [4], [19]–[27].

In 2001, Zang reported a genetic model of tumor progression in meningiomas [8]. It was shown that the loss of chromosome 22 and the deletion of one short arm of one chromosome 1 was followed by complete or partial loss of additional chromosomes, in particular of chromosomes 6, 10, 14, 18, 19 [3], [8], [21], [24], [28]–[33]. Based on these findings, we applied a new mathematical model for tumor genesis to a group of 661 cytogenetically characterized meningioma patients, including 53 patients with tumor recurrences [3]. This model allows for estimating the probability of recurrences in meningiomas based on their genetic aberrations. The findings prompted us to investigate the loss of the short arm of one chromosome 1 in meningiomas in daily clinical practice.

Since the publication of Ishino and coworkers in 1998 the fluorescence in situ hybridization (FISH) technique has provided new insights into interphase cytogenetics and is applicable now to clinical practice. Therefore the time-consuming cell culture for conventional chromosome analysis is not further necessary. Instead of paraffin embedded tumor samples, also fresh tumor samples that are speckled on microscope slides can be used.

In the current study we examined 124 meningioma samples out of 105 prospective meningioma patients for the partial loss on chromosomes 1p, 9p and 22q, using double-target iFISH. The first aim of this study was to analyze if the iFISH results predict malignant behavior and recurrence of meningiomas as well as conventional cytogenetic analysis. The second aim of this study was to examine the promoter hypermethylation of *TIMP3* and *p16* in meningiomas in order to evaluate the impact of these markers for the biological behaviour of meningiomas.

## Materials and Methods

### Patient population

We performed a prospective study on 124 tumor samples from 105 meningioma patients [76 women and 29 men] operated at the Department of Neurosurgery, Saarland University, between January 1997 and December 2010. The average age of the overall patient population at the date of first surgery was 57.2 years [SD  = 13.3 years], for female patients 58.1 years [SD  = 12.0 years], and for male patients 54.8 years [SD  = 16.3 years]. Written informed consent was obtained from each patient participating in the study.

### Follow up

Patients were examined in the neurosurgical outpatient department of the Saarland University, either within routine follow-up or upon appearance of neurological symptoms. A recurrence was defined as new evidence of tumor in CT or NMR after previous complete extirpation (Simpson grades 1 and 2) [43]. The Simpson grade 2 was established on the basis of the operation report and the postoperative CT or NMR investigation.

### Tumor extirpation

Complete surgical extirpation of the tumor was defined as Simpson grades I and II. This corresponds to a macroscopically complete tumor resection with bipolar coagulation of the dura insertion.

### Tumor histology

Meningioma grade was assessed by a combined histologic [21], [22], [32], [44]–[46] and morphometric approach on routinely HE and Ki-67/Feulgen stained formalin-fixed, paraffin-embedded tissue sections [21]. The 105 meningiomas comprised 63 tumors of WHO grade I, 35 tumors of WHO grade II, and 7 tumors of WHO grade III.

All tumors were classified according to the WHO classification of tumors of the nervous system of 2007 [46].

In total 124 different probes were investigated from the 105 patients. In 91 tumors (86.6%), only one surgical procedure was performed, in 10 patients (9.5%) 2 surgery procedures, in 3 patients (2.9%) 3 operations due to recurrence, and in 1 patient 4 surgical approaches was necessary.

Table 1 and 2 contains a breakdown of age, sex, WHO grade, chromosomal aberrations, and methylation status of *TIMP3*.

**Table 1:** Correlation between clinical variables and WHO tumor grade in meningiomas.

Tumor grade	WHO I	WHO II	WHO III
No of patients	62 (59.05%)	35 (33.33%)	8 (7.62%)
Age in years (mean ± SD)	56.31±13.61	58.76±12.72	57.13±14.60
Gender (Females/Males)	50/12	24/11	2/6
Recurrences (%)	4 (6.45%)	11 (31.43%)	6 (75.00%)
Mean follow up time (years)	3.23	5.71	8.06
Timp3	6 (9.68%)	7 (20%)	1 (12.50%)

**Table 2:** Correlation between clinical variables and deletion of 1p36.

Tumor grade	Deletion of 1p36	No Deletion of 1p36
No of patients	39 (37.14%)	61 (58.10%)
Age in years (mean ± SD)	59.72±13.22	55.99±13.42
Gender (Females/Males)	24/15	47/14
Recurrences (%)	14 (13.33%)	7 (6.67%)
Mean follow up time (years)	4.53	4.41
Histological type	No. of tumors	
Common typeWHO grade I	18	41
AtypicalWHO grade II	14	19
AnaplasticWHO grade III	7	1

### FISH analysis

Tissue specimens from tumors were obtained freshly after surgery and dapped on clean microscope slides previously silanized. After Delauney fixation for 30 sec, slides were stored at −20°C until further FISH analysis. The slides were treated with RNAse for thirty minutes at 37°C and placed three times in 2xSSC for five minutes at RT. Cells were digested in 100 ml 0,01 M HCL with 10 mg pepsin (Serva Heidelberg, Germany) for 1 min and 45 sec at 37°C. Slides were dipped in 1xPBS for 5 min, 4%PFA/1xPBS for 10 min for fixation and 1xPBS for 5 min. They were then dehydrated in 70%, 80%, 95% ethanol and air dried. Dual-probe hybridization was performed using locus-specific probes for 1p36, 22q11 and 9p21 (Abbott, Germany and Metasystems, Germany). Target and probe were separately denatured for 5 minutes at 73°C. Target slides were dehydrated in 70%, 80%, 95% ethanol. Then probes were pipette on slides and incubated overnight at 37°C in a humidified chamber. Stringency wash was performed in 0.4xSSC/0.3%SDS for 2 minutes at 73°C and 2xSSC/0.1% SDS at RT. Finally, slides were counterstained with DAPI antifade (Vectashield, Vector Laboratories).

**Figure 1:**
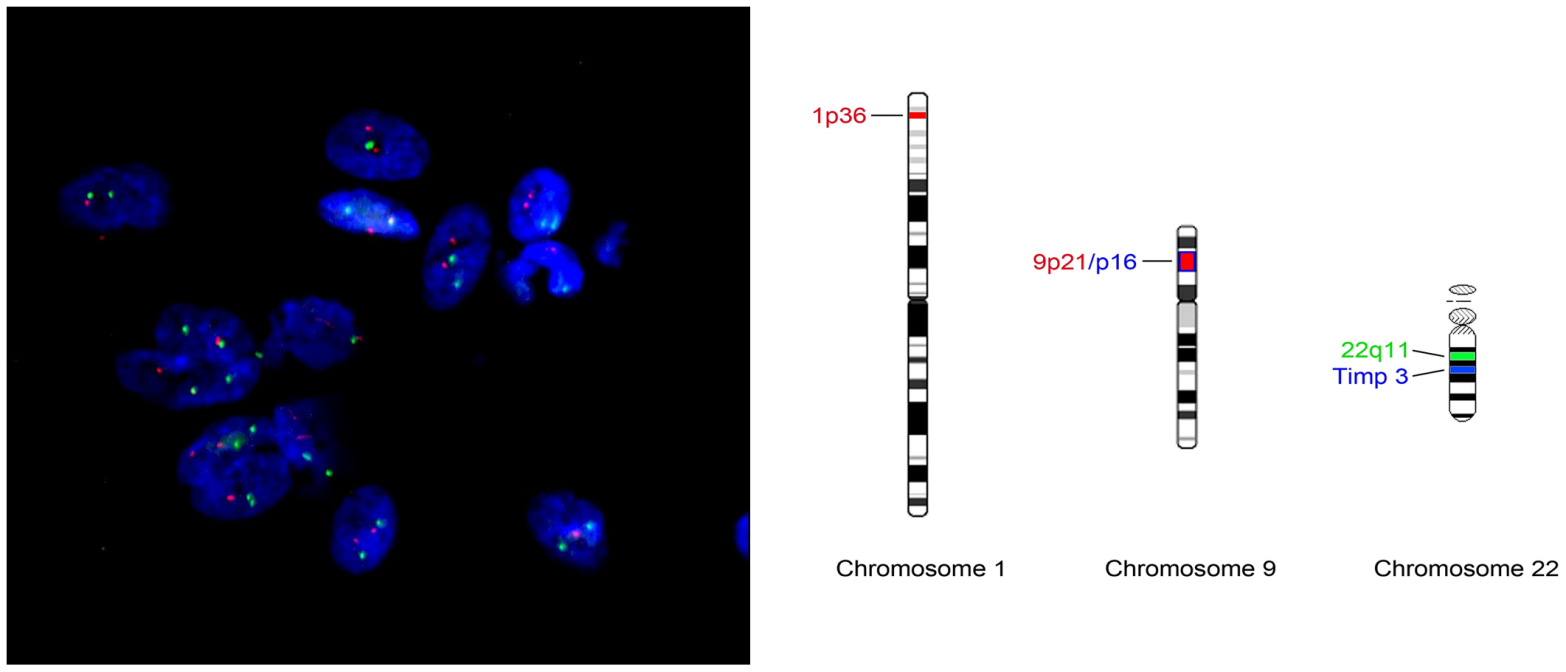
**Figure 1a:** Two color FISH for chromosome 1p36/22q11 (Metasystems). **Figure 1b:** Probe map for dualcolor probe 1p36/9p21 and 22q11 (Metasystems).

At least 200 non-overlapping nuclei per sample were counted for evaluation according to Hopman-criteria [47], using an Olympus AX70 fluorescence microscope. Figures 1a and 1b illustrate the mapping of FISH probes.

Cut-offs for alterations was determined by comparison with normal human lymphocytes control samples at 10% for deletions of 1p36, 22q11 and 9p21.

### DNA extraction

Extraction of genomic DNA from snap frozen tumor tissue was performed with standard methods with samples more than 0.3 g and QIA DNA Mini Kit for samples with less than 0.3 g (QIAGEN, Hilden). DNA-Isolation from peripheral blood leukocytes was performed according to standard protocols [48].

### Methylation analysis with MS- PCR and sequencing

**Figure 2:**
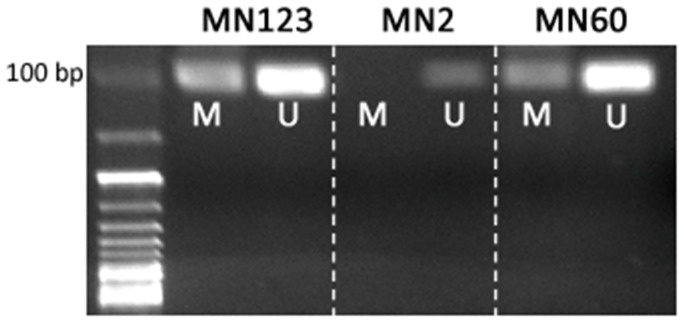
Demonstration of PCR products of *TIMP3* in gelelectrophoresis: MN 123 and MN 60 =  patients with positive methylation status of *TIMP3*. MN 2 =  patient without methylation.

Sodium bisulfite treatment of 500 ng DNA was performed with the EZ Methylation Gold™ Kit (Zymo Research Corporation) according to the manufacturer's protocol. PCR analyses were performed with 70 to 80 ng of the bisulfite-modified DNA as template using 0.2 μl (5 U/μl) of HotStar Taq DNA Polymerase (QIAGEN, Hilden) in 1× Qiagen PCR-buffer with 0.25 mmol/l of each dNTP and 0.1 μmol/l of each primer. The primers used in this study are described in the literature by Herman et al. [49] (p16) and Righini et al. (TIMP3) [50]. We used 0.5 μl MgCl2 (25 mM) (Quiagen) and 1.25 μl DMSO (5%v/v) for MS-PCR of TIMP3 as PCR-additive. MS-PCR with p16-primers was carried out for 37 (unmethylated specific analysis)/40 cycles (methylated specific analysis); 40 cycles TIMP3-MS-PCR with specific primers for both methylated and unmethylated alleles. MS-PCR was carried under the following conditions: 15 min initial denaturation at 95°C followed by 40 cycles of 1 min denaturation at 95°C, 1 min primer-annealing at 65°C (p16)/59°C (TIMP3), 1 min extension at 72°C and a final extension of 1 min at 72°C. As a negative control we added water in place of DNA and as a positive control we used Universal Methylated Human DNA (Zymo Research). PCR-products were separated on 2–3% agarose gels, than stained with ethidium bromide. Bands were visualized under UV (UV-transilluminator Wealtec, Sparks, USA), recorded (camera CU105M, 8030102 Rainbow, Japan) and detected (DeVision G, Vers. 2.0; Devon Science Tec GmbH, Hohengangern, Germany). Exemplary products of gelelectrophoresis are shown in Figure 2.

Also, we analysed methylation status of *p16* and *TIMP3* by sequencing the regions of CpG-Islands as described in previous studies [17], [51]. The PCR products were extracted and subjected to cycle sequencing with BigDye Terminator sequencing kit and analysed on an ABI 3730 automated DNA capillary sequencer (Genterprise, Mainz, Germany).

### Statistics

Comparison of survival times between groups defined by methylation status was performed by Kaplan-Meier curves and with two-sided log rank tests. Univariate and multivariate Cox regression analysis was performed to identify significant predictors for survival and tumor recurrence. Stepwise backward selection based on the AIC (Akaike Information Criterion) was used for variable selection for the multivariate Cox model. Effects in all models were quantified by hazard ratio estimates with corresponding 95% confidence intervals.

All p-values were calculated with two-sided tests. Median progression free survival rates were calculated using the Kaplan-Meier method.

### Ethics Statement

We obtained ethical approvel from local ethics committee for “The role of high risk of local recurrence in meningioma as indicated by the genetic progression score” (ethical approval No. 178/07) involving both, Department of Human Genetics, Saarland University and Neurosurgical Clinic, Saarland, University Medical School.

Written informed consent was obtained from each patient participating in the study.

## Results

We performed a prospective study on 124 tumor samples from 105 meningioma patients. The sex ratio was 2.6:1 in favor of females (76 women and 29 men). The average age of all patients at the date of first surgery was 57.2 years, and females were slightly but non-significant older on average (58.1 years vs. 54.8 years), see Table 1 and Table 2.

In 91 tumors (86.6%), only one surgical procedure was performed, in 10 patients (9.5%) 2, in 3 patients (2.9%) 3, and one patient had 4 times a tumor resection.

**Figure 3:**
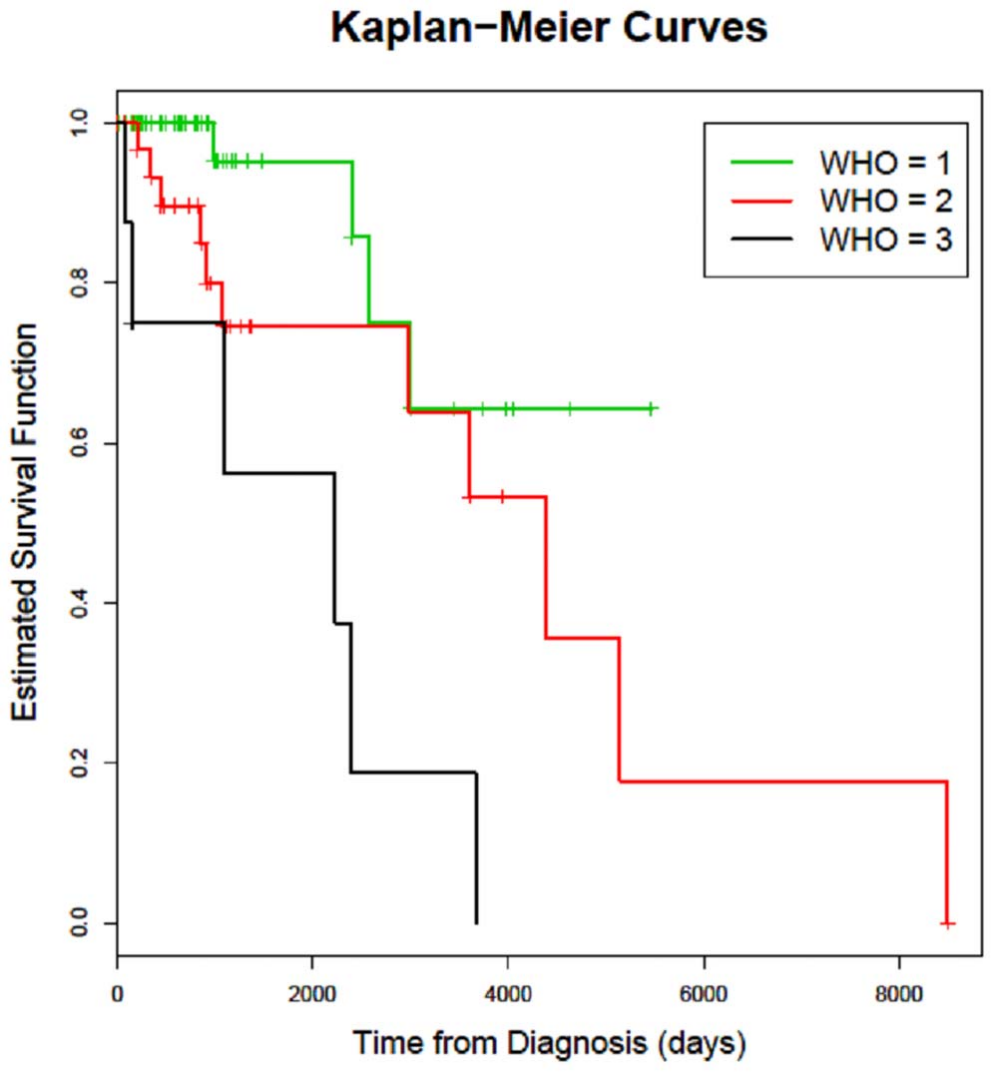
Kaplan-Meier survival curves for times to recurrence of meningioma patients; patients are split into 3 subgroups according to WHO grades.

The histological results showed in 62 cases (59.05%) a WHO grade I tumor, in 35 cases (33.33%) a WHO grade II and in 8 cases (7.62%) a WHO grade III. The WHO grade was significantly correlated with tumor recurrence (p = 0.0026; Table 1; Figure 3).

In 59% of the investigated cases we detected chromosomal aberrations by FISH analysis: loss of the long arm of chromosome 22 in 48.6% (51/105), loss of the chromosomal region 1p36 in 35.2% (37/105) and loss of the chromosomal region 9p21 in 6.67% (7/105).

Breaking down the FISH results to the respective WHO grading: monosomy 22 was detected in 45.2% (28/62) of WHO grade I, 48.6% (17/35) in grade II and 75% (6/8) in grade III meningiomas. Univariate as well as multivariate Cox regression analysis revealed no correlation with tumor recurrence (p = 0.771; p = 0.2, Table 3).

**Table 3:** Univariate and multivariate Cox-regression analysis.

Variable	Univariate analysis			Multivariate analysis		
	HR	95% CI	p-value	HR	95% CI	p-value
Sex (female)	0.572	0.227–1.442	0.237	6.036	0.876–41.610	0.0679
Age	1.000	0.999–1.000	0.891	0.999	0.999–1.000	0.2497
WHO grade II	3.002	0.932–9.673	0.065	3.367	0.948–11.962	0.0606
WHO grade III	10.198	2.838–36.645	<0.001 (*)	9.588	1.104–83.296	0.0404 (*)
TIMP3b	0.851	0.246–2.941	0.799	0.146	0.021–0.979	0.0475 (*)
p16	0.769	0.247–2.390	0.650	0.996	0.283–3.450	0.9944
1p36	4.962	1.952–12.611	<0.001 (*)	11.005	2.261–53.550	0.0030 (*)
22q	1.143	0.465–2.813	0.771	0.465	0.144–1.516	0.2041
9p	3.352	1.187–9.463	0.0224 (*)	1.881	0.383–9.226	0.4363

Results for univariate and multivariate Cox regression models. For every variable, the estimated hazard ratio HR and its corresponding 95% confidence interval are given, as well as the p-value when testing HR = 1. P-values below 0.05 are marked with a star (*). The categorical variable WHO grade is treated as factor variable, thus HR refers to the comparison of WHO grade II and WHO grade III, respectively, to WHO grade I.

**Figure 4:**
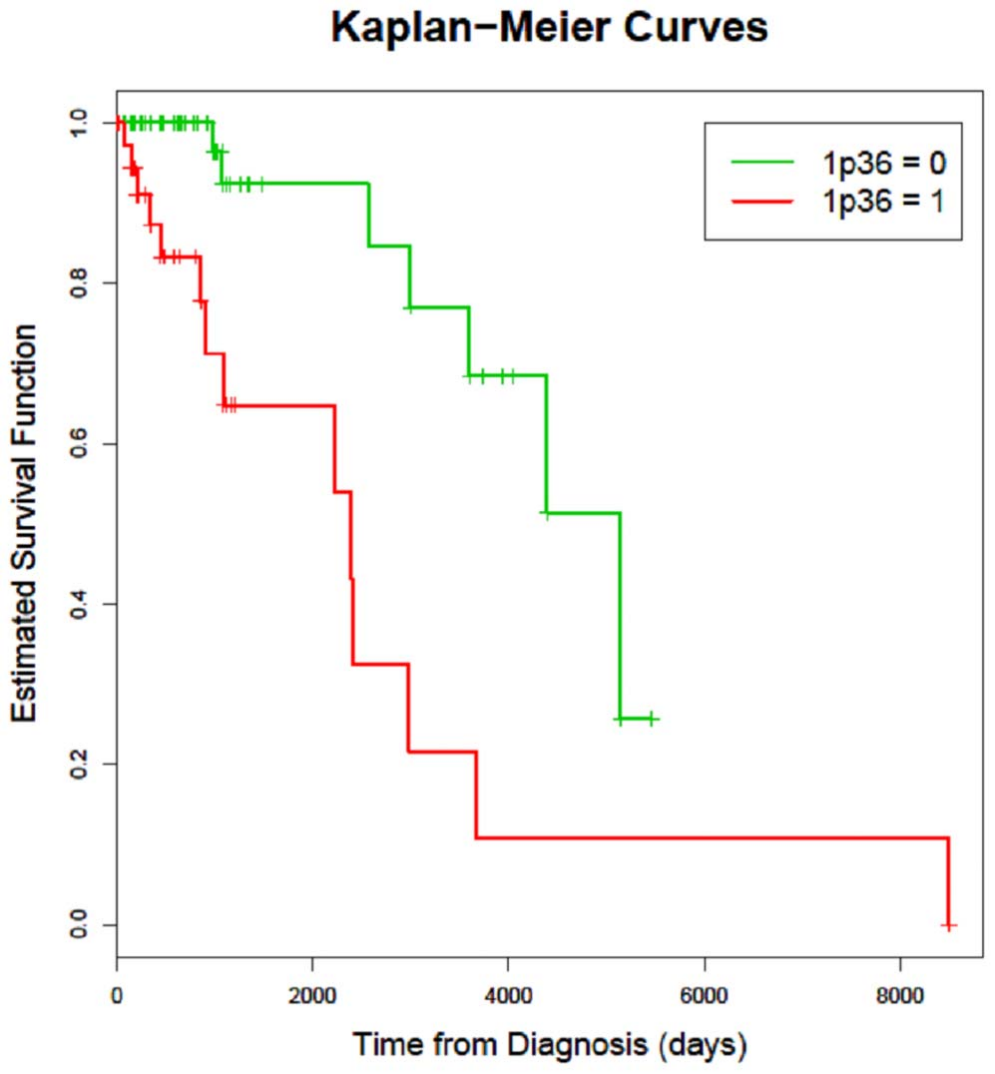
Kaplan-Meier survival curves for time of recurrences of meningioma patients; patients are split into 2 subgroups according to Deletion of 1p36.

In WHO grade I meningiomas loss of 1p36 was detectable in 27.42% (17/62), in grade II in 27.1% (13/35) and in anaplastic meningiomas in 87.5% (7/8) of cases. Table 2 shows the number of recurrences and Figure 4 the time to recurrence, stratified by deletion of the short arm of one chromosome 1 (deletion of 1p36). Recurrences after complete tumor extirpation were observed in 21 of the 105 cases (20%). The deletion of 1p36, was found to be highly statistically significant predictive for the recurrence of a given meningioma in univariate Cox-regression analysis (p<0.001; Table 3) as well as in multivariate Cox-regressions analysis (p = 0.0030 before variable selection, Table 3, and p = 0.0026 after variable selection based on AIC, Table 4). Tumors with deletion 1p36 showed a recurrence rate of 25.9% (N = 14), tumors without this deletion showed a recurrence rate of 11.5% (N = 7).

**Table 4:** Multivariate Cox-regression analysis with variable selection by AIC criterion.

Variable	Multivariate analysis		
	HR	95% CI	p-value
Sex (female)	4.727	0.834–26.779	0.0792
WHO grade II	3.239	0.894–11.733	0.0735
WHO grade III	10.347	1.325–80.798	0.0259
TIMP3b	0.175	0.029–1.043	0.0557
1p36	10.447	2.266–48.164	0.0026
22q	0.411	0.135–1.251	0.1174

Results for multivariate Cox regression models with variable selection. For every variable, the estimated hazard ratio HR and its corresponding 95% confidence interval are given, as well as the p-value when testing HR = 1. The categorical variable WHO grade is treated as factor variable, thus HR refers to the comparison of WHO grade II and WHO grade III, respectively, to WHO grade I. The full model before variable selection included the additional variables age, p16, and 9p which were eliminated by stepwise backward selection with standard AIC (Akaike Information Criterion) as model selection criterion. All estimates and p-values in the table are close to the corresponding values before variable selection (see Table 3).

Only 1.6% (1/62) of WHO I meningiomas and 5.7% (2/35) of WHO II meningiomas had a deletion of 9p21. On the other hand 50% (4/8) of anaplastic meningiomas showed this deletion (see also Table 3).

Hypermethylation of *TIMP3* was found in 13.3% (14/105) of all cases. Breaking down the methylation results by the WHO grade, 6 cases (9.68%) belonged to WHO grade I, 7 cases (20%) to WHO grade II and 1 case (12.5%) to the anaplastic meningioma grade III (Table 1).There was an increasing rate of *TIMP3* hypermethylation from grade I (9.68%) to grade II (20%). However due to the small number of cases of anaplastic meningiomas these results turned out to be not significant (p = 0.308; Table 1).

Comparison of *TIMP3* hypermethylation between the time to recurrence of the given meningioma and the methylation status revealed in univariate Cox regression analysis no significant correlation (p = 0.799; Table 3). Interestingly the multivariate Cox regression analysis of the hypermethylation of the promoter *TIMP3* and the time to recurrence turned out to be significant (p = 0.0475; Table 3).

The methylation of the tumor suppressor gene *p16* was detected more frequently, but it was not associated with recurrence, higher grade, or chromosomal aberrations. Neither age nor sex had significant influence on tumor recurrence, with regard to the *p16* methylation status.

In Table 5 the interaction of the investigated molecular markers which each other is shown. Only two marker pairs are significantly correlated: the deletion of chromosomal region 1p36 with the deletion of 22q (p = 0.0005), and the deletion of 1p36 with the deletion of 9p (p = 0.034). For all other markers no significant correlation could be observed. Since the deletions of 1p36 and 22q are highly correlated, but only deletion of 1p36 is associated with meningioma recurrence, we performed an analysis including only patients with deletion of 1p36. Comparing in this subgroup patients with and without deletion of 22q no significant difference between recurrence times was observed (p = 0.96, log rank test).

**Table 5:** Correlations between binary molecular markers.

	TIMP3b	p16	X1p36	X22q	X9p
**TIMP3b**	1-	−0.085 (0.5117)	0.029 (0.7760)	−0.128 (0.2541)	0.114 (0.2572)
**p16**	−0.085 (0.5117)	1-	−0.003 (1)	0.007 (1)	0.035 (0.6622)
**X1p36**	0.029 (0.7760)	−0.003 (1)	1-	0.358 (0.0005)	0.263 (0.0134)
**X22q**	−0.128 (0.2541)	0.007 (1)	0.358 (0.0005)	1-	0.028 (1)
**X9p**	0.114 (0.2572)	0.035 (0.6622)	0.263 (0.0134)	0.028 (1)	1-

Correlations between pairs of all binary molecular markers and corresponding statistical significance values. In each cell, the top entry gives the phi coefficient between the corresponding two markers. The phi coefficient is a measure of association for two binary variables and equals the Pearson correlation coefficient in this case. The bottom entry (in brackets) gives the p-value of Fisher's exact test for the corresponding two markers, where a small value indicates a significant association. Only two pairs are significantly correlated, namely X1p36 and X9p (correlation 0.263, p-value 0.0134) and X1p36 and X22q (correlation 0.358 and p-value 0.0005). All other correlations are smaller than 0.13.

## Discussion

Grading of meningiomas has always been controversial. Obviously, the biological behaviour of meningiomas cannot be accounted by histological parameters alone [3], [4], [21], [31]. There is agreement in the literature that radical surgical extirpation is correlated with good prognosis [13], [14], [16], [22], [32], [52]. We therefore included only patients in our study whose tumor had been macroscopically completely removed, corresponding to Simpson grades I and II [43].

It is well known that females are affected far more frequently by meningiomas than males [9], [13], [21], [32], [38]. This observation was confirmed in our patients with a ratio of 2.6:1. This shift confirms earlier reports [3], [4], [5], [38], [46], [53].

In a panel of 105 meningiomas covering the different WHO grades, we assessed the methylation status of the tumor suppressor genes *TIMP3* and *p16* as related to their chromosomal aberrations (Table 1, 3).

The gene for the tissue inhibitor of metalloproteinase 3 (*TIMP3*) on 22q12.3 has been described as a tumorsupressor gene in different tumor entities [9], [13], [15].

In previous studies by Bello [54] and Lomas [55] hypermethylation of the *TIMP3* gene resulting in silencing the tumorsupressor gene was more frequently detected in meningiomas with loss of 1p. Bello and coworkers found a correlations with loss of the genes *THSB1* (47%), *TIMP3* (24%), *p73* (33%), *p14ARF* (23%) and *p16* (23%). Also meningiomas with loss of 22q demonstrated a higher methylation status in this study [54]. Van Tilborg and co-workers reported hypermethylation of *p16* in 62% of meningiomas with LOH of *NF2* (located on 22q12.2) in contrast to the normal karyotype [56].

While Bello and colleagues [54] detected hypermethylation of *TIMP3* 5′-CpG island region in about 24% of meningiomas, Liu [57] could not validate these findings. Barski [9] assessed *TIMP3* hypermethylation with MS-PCR and direct sodium bisulfite sequencing and revealed *TIMP3* methylation in 67% of anaplastic meningiomas but less in atypical (22%) or benign meningiomas (17%).

In our present study we found that the rate of patients with *TIMP3* methylation increases from 9.68% in grade I to 20% in grade II meningiomas. However, due to the low rate of the *TIMP3* methylation in grade III meningiomas (1/8 caseş12.5%) in our study, overall TIMP3 methylation was not significantly correlated with tumor grade (p = 0.308, Fisher test).

Interestingly, the clinical follow-up data of the investigated meningioma patients revealed in the multivariate Cox-regressions analysis, that the methylation of the *TIMP3* gene is significantly associated with a shorter time to recurrence (p = 0.0475; Table 3). These data require further investigations about the mechanism of inactivation of *TIMP3* in meningiomas relating to a more aggressive clinical behaviour, which is discussed in the literature [9], [12], [14], [53].

Divergent results reported in the literature [9], [28], [54], [57] might stem from the different techniques and primer sites. Bello and Barski used different CpG islands as regions of interest for sequencing [9], [54]. As a result of these divergent findings with the failure of hypermethylation detection by Barski and colleagues at priming site -62 - 59 as well as the low hypermethylation rate in our sequencing analysis with primer site -50+2 we introduced the MS-PCR of a previously unaddressed, more distal region (-21 -18) for the methylation status analysis in our meningiomas [9], [54]. Therefore in our study the MS-PCR of the *TIMP3* region was more sensitive and yielded more reliable results of methylation status. This is in line with previous reports. Generally, MS-PCR is known to be less prone to false positive results than sequencing [49]. Based on these finding all further analyses of methylation status were done by MS-PCR.

*TIMP3* maps on 22q12, a chromosomal region that gets lost in about 50% of sporadic meningiomas [30]. This is consistent with previously reported data by Barski [9]. However, in our study *TIMP3* methylation did not occur exclusively in meningiomas with deletions on 22q. This controversy is maybe caused by the retrospective panel of only 39 meningiomas investigated by Barski and colleagues.

Interestingly, loss of chromosome 1p - as an important genetic prognostic marker in meningiomas – and also loss of 9p – a typical chromosomal aberration in anaplastic meningiomas [2] – did not accompany hypermethylation of *TIMP3*.

The hypermethylation status of *p16* did not coincide with chromosomal aberrations or high incidence of recurrences. LOH of 9p is very rare in meningiomas (6% of our patients) and was not concordant with the hypermethylation status of *p16*. Furthermore our results did not confirm the association of hypermethylation of *p16* in meningiomas with LOH of the *NF2* gene from the previous study by van Tilborg [56].

In Table 5 the dependence between the investigated molecular markers is analysed. Only two marker pairs are significantly correlated, deletion of chromosomal region 1p36 with deletion of 22q (p = 0.0005) and deletion of 1p36 with deletion of 9p (p = 0.034). For all other marker pairs no significant correlation could be found.

The significant association of the deletion of 22q with the deletion of one short arm of one chromosome 1 is in line with our previously published results describing the clonal cytogenetic evolution of meningiomas [3].

Although Barski et al. found in their study that in 20 of 39 investigated meningiomas the methylation of the *TIMP3* status was associated with a micro deletion on the long arm of chromosome 22, we could not confirm these results. However, it must be given consideration that the monosomy 22 was ascertained by classic cytogenetic analyses in our study, whereas Barski and coworkers used microsatellite analyses using primers who span the *TIMP3* region on chromosome 22 [3], [9].

After chromosome 22 anomalies, aberrations of the short arm of one chromosome 1 are the most frequent alterations detected by cytogenetic analysis of meningiomas [3], [4], [8], [21], [24], [28]–[33]. In a former study, we introduced the GPS established by cytogenetic analysis, as a marker for tumor progression. The GPS of a tumor is defined as the estimated average waiting time of its observed genetic pattern in a timed oncogenetic tree model. Using Cox regression analysis we demonstrated that for meningiomas the GPS has prognostic value with respect to clinical outcome and recurrence (p<0.001) [3].

In the present study we confirmed these previous results using the high resolution FISH-technology (iFISH) [3]. In univariate Cox regression analysis (p<0.001) as well as in multivariate Cox regression analysis (p = 0.0030 before variable selection and p = 0.0026 after variable selection based on AIC). deletion of 1p36 serves as an independent marker for the clinical behaviour of the given tumor.

In Figure 4 it is shown, that a deletion of 1p36, detected by iFISH in a given meningioma, can perfectly be used to identify a subgroup of tumors with poorer prognosis with respect to time to recurrence after surgery. Thus the genetic classification allows a prognostically significant distinction between low-risk and high-risk meningiomas at the time of primary surgery. It can be expected that a combination of both histopathological and cytogenetic descriptions of meningiomas could result in even improved prognostic accuracy [21], [23], [40], [42], [53].

Our results are in agreement with former cytogenetic investigations which indicated that the deletion of the distal part of the short arm of a chromosome 1 [1p-] is associated with progression in meningiomas [3], [4], [8], [21], [24], [28−33], [46], [58], . According to the above-mentioned findings, the deletion of chromosome 1p was ascertained to be an early and crucial event in the progression in meningiomas. By fitting multivariate Cox regression models we also found that for meningiomas the deletion of one short arm of one chromosome 1 has a very high impact concerning the biological behaviour in terms of recurrence and malignant behaviour (p = 0.00297,Table 3).

In conclusion, in this study we used iFISH that is easily applicable to clinical practice because it is not very time-consuming and can be applied to various clinical materials, including paraffin embedded tumor samples or dapped slides. Consequently, due to the common feasibility of the iFISH method a multimodal approach to meningioma grading, which is in our opinion the most promising for identifying meningiomas with an increased tendency to recur, is now available for almost all patients.

The *TIMP3* inactivation by methylation seems to be also involved in meningioma progression, at least it is associated with a shorter time to recurrence using the multivariate Cox-regression analysis (p = 0.0475).

Based on the results of this study, the deletion of the short arm of one chromosome 1 is an independent prognostic factor which correlates significantly with increased risk of recurrence. After initial speculation on the role of 1p deletion for tumor recurrence [28] the importance of this aberration besides monosomy 22, 14 and 10 for the development of atypical and anaplastic meningiomas [18] and for progression from typical to atypical meningioma [3], [8] was pointed out.
